# Optimal Values of Body Composition for the Lowest Risk of Failure in Tabata Training's Effects in Adolescents: A Pilot Study

**DOI:** 10.1155/2021/6675416

**Published:** 2021-02-24

**Authors:** Jarosław Domaradzki, Andrzej Rokita, Dawid Koźlenia, Marek Popowczak

**Affiliations:** ^1^Department of Biostructure, University School of Physical Education in Wroclaw, Al. I.J. Paderewskiego 35, 51-612 Wroclaw, Poland; ^2^Department of Team Sports Games, University School of Physical Education in Wroclaw, Al. I.J. Paderewskiego 35, 51-612 Wrocław, Poland

## Abstract

**Background:**

The optimal body mass index (BMI) and fat mass index (FMI) values for a positive change or the lowest risk of no positive change after high-intensity interval training (HIIT) using the Tabata protocol remain unclear. This study is aimed at establishing these optimal BMI and FMI values for the lowest risk of failure of aerobic performance in adolescents.

**Methods:**

A 10-week HIIT programme was introduced into the physical education of 73 students. BMI was calculated using height and weight. Bioelectrical impedance analysis measured body fat, and the InBody apparatus generated the FMI. Based on BMI and FMI, the participants were divided into four groups. Pre- and post-HIIT intervention analyses were carried out using the Harvard step test, which was used to determine the physical efficiency index (PEI).

**Results:**

The Youden index confirmed that the risk of no positive effects in PEI was the lowest for the second BMI interval (19.01-22.00 kg/m^2^) and FMI *Q*_2−3_ (7.96-8.91 kg/m^2^). The optimal BMI value for the lowest risk of no change in PEI was 20.60 kg/m^2^, and the optimal FMI value was 8.84 kg/m^2^.

**Conclusion:**

A comparison of the two indices shows that FMI had stronger effects on PEI than BMI. In addition, the model obtained for FMI had higher accuracy. Identifying at-risk individuals, those in need of improving health-related fitness (H-RF), and those with a low risk of poor H-RF allows for efficient planning of individual intervention services and training programmes.

## 1. Introduction

Normal body weight and body mass index (BMI) are primary morphological determinants of health [1]. Excess body mass is the main nutritional health-related problem of modern societies, predominantly affecting the health of children and young people [2]. Furthermore, its persistence into adulthood affects the health of working age adults and increases the risk of cardiovascular disease, diabetes, and metabolic disease [3, 4]. The number of overweight and obese boys and girls aged 5-19 years has increased by approximately tenfold between 1975 and 2016 [5].

An effective strategy to battle obesity and overweight among children and young people is to introduce physical activity programmes and/or increase the amount and intensity of physical education in schools [6]. One promising method is high-intensity interval training (HIIT) [7]. The effects of HIIT programmes are manifested in improved physical performance, a reduction in circulatory-respiratory disease biomarkers, a lower level of insulin resistance, a lower body mass index (BMI), a lower body fat percentage (BFP), and better physical fitness [8, 9]. Tabata training is one of the most popular HIIT methods. This exercise method was originally developed for cycling [10]. The basic format includes several circuits (typically 7 or 8) of 20 s of exertion followed by 10 s rest [11], including various types of physical exercises.

The effectiveness of a HIIT programme is commonly assessed through BMI changes in subjects after training. However, despite the frequent use and popularity of BMI in assessing overweight and obesity, its accuracy and usefulness has been criticized because BMI does not account for true body fatness status. BMI has been shown to be insensitive to people with increased body fat and low body muscle and to have some limitations in assessing the risk of obesity-related diseases [12, 13]. In some studies, the effects of multiweek HIIT programmes were observed in overweight young people and in those with normal BMI; the results indicated improvements only in overweight individuals [12, 14, 15].

Another limitation of BMI is in determining the usefulness of different body composition measures and their value in predicting metabolic disease. An emerging alternative for assessing obesity and overweight is the fat mass index (FMI) [13]. FMI is the ratio of fatty tissue mass to body height. The FMI has recently gained significance, owing to several factors including the similarity of its calculation method to that of BMI [16, 17]. FMI is calculated using the body fat mass component from bioelectrical impedance analysis (BIA). Owing to the high reliability of BIA and its high correlation with the results of dual-energy X-ray absorptiometry (DXA) in body composition evaluation, body fat percentage (BFP) has become widely used [18, 19].

BMI is associated with aerobic, anaerobic, and motor performance [12, 14, 15]. Moreover, recent studies indicate that the effects of HIIT programmes on body composition and physical efficiency may depend on BMI status [8, 12]. However, it is not known whether the effects of HIIT are the same for underweight (<18.5 kg/m^2^), normal weight (18.5-24.99 kg/m^2^), and overweight (>25 kg/m^2^) individuals. Furthermore, the fatness level expressed by FMI is rarely used in such analyses. The better predictor of HIIT effects between BMI and FMI indices, particularly during aerobic performance, is not known. Furthermore, it has not been explicitly determined what values of BMI accommodate positive changes or the lack of changes after the HIIT. The aim of this study was to establish the optimal BMI and FMI values for the lowest risk of failure (no changes in physical efficiency) in aerobic performance in adolescents after a 10-week HIIT programme. Secondly, a comparison between the accuracy of the BMI and FMI models was desired. Ultimately, the most favourable body composition suitable for the positive changes in aerobic performance, resulting from the HIIT programme was determined.

## 2. Materials and Methods

### 2.1. Participants

The participants were 73 adolescent students (31 boys: 16.24 ± 0.31 (15.51-16.70) and 42 girls: 16.12 ± 0.39 (15.30-16.70) from a secondary school in Wroclaw, Poland. The students and their parent/guardian were informed about the objectives of the study, that participation was voluntary, and that they could opt out at any time. The school principal, parents, and study participants gave written informed consent prior to participation. The Ethics Committee of the University of Physical Education in Wroclaw approved the project (ECUPE no: 19/2019). The study was conducted in accordance with the Declaration of Helsinki by the World Medical Association for research with humans. The project also met the ethical standards for sports medicine.

### 2.2. Measurements

Height was measured using a Swiss anthropometer (GPM Anthropological Instruments, DKSH Ltd., Switzerland). Body weight and fat mass were measured using the InBody 230 based on the BIA method (InBody Co. Ltd., USA). All the measurements were done by trained staff. The BMI was calculated from the formula:

BMI = body weight (kg)/body height^2^ (m^2^).

The FMI was calculated from a similar formula:

FMI = body fat mass (kg)/body height^2^ (m^2^).

Participants were divided into four groups, each according to BMI and FMI values. These groups were used as the intervals for receiver operating characteristic curve calculations.

The adolescents were grouped based on BMI (<19 kg/m^2^, 19.01-22.00 kg/m^2^, 22.01-25.00 kg/m^2^, and >25 kg/m^2^). The first group (≤19 kg/m^2^) was chosen as the reference group to calculate the Youden index. In addition, their FMI values were also divided into four equal groups according to quartiles (below *Q*_1_, *Q*_1−2_, *Q*_2−3_, and over *Q*_3_). The first group (below *Q*_1_) was chosen as the reference group to calculate the Youden index.

### 2.3. Procedures

The effect of the HIIT programme based on the Tabata procedure on aerobic performance was assessed [20]. The students carried out a 14-minute HIIT programme (in the form of a movie) during one of their three physical education (PE) lessons per week. The remaining PE lessons were conducted in accordance with the PE curriculum adopted by the school for the first year students. The HIIT programme lasted 10 weeks [21]. Each week, a standardized 10 min warm-up consisting of 5 min of slow jogging followed by 5 min of stretching (dynamic and static) of the major muscle groups was performed followed by a 14-minute Tabata training. The Tabata training was divided into three sessions, each lasting 4 min. Each session consisted of eight cycles of two exercises. Each cycle started with a maximum intensity exercise lasting for 20 s, in which the participant was motivated to perform as many repetitions as possible of a given exercise involving large muscle groups of the entire body, followed by a 10 s active rest in the form of a low-intensity exercise. The cycles were repeated without rest. There was a 1 min passive rest period between each session during which no exercises were performed. After the Tabata training, flexibility and relaxation exercises were performed for several minutes. All the exercises were prepared by the authors (for the purpose of the experiment), recorded, and played during the PE lesson on a screen to ensure that the times of exercise and rest were implemented accurately.

Aerobic performance was measured indirectly using the Harvard step test with a step box height of 41.3 cm. This testing procedure revealed high reliability, (ICC ≥ 0.95) [22]. The participants went up and down the step box at a pace of 30 cycles per minute with a metronome set at 120 beats per minute (bpm). The exercise was performed for 300 s or less if the participants were compelled to stop owing to exhaustion, which was measured as the time of effort (*L*). After the test (during 1.5 min period after finishing), heart rate (*p*) was measured. The physical efficiency index (PEI) was calculated using the short formula [23].

(1) $$\begin{array}{c} \text{PEI}=\frac{100\times L}{5.5\times p}. \end{array}$$

The PEI values before and after the intervention were calculated and compared. A higher PEI after the intervention was recorded as a positive change, whereas the same or lower PEI value before the intervention was recorded as a negative change. This suggests that the effects of the intervention were recorded on a dichotomous scale. All the measurements were performed by qualified staff. The tests took place in the certified Laboratory of the Physical Education Faculty at the University School of Physical Education in Wroclaw (ISO number: PN-EN ISO 9001 : 2009 and Certificate number: PW-48606-10E).

### 2.4. Statistical Analysis

The relationships between sex and BMI, FMI, and PEI were assessed using the chi-square test (*χ*^2^). The linear relationships between the preintervention PEI and BMI and PEI and FMI were evaluated using multiple regression analysis. Receiver operating characteristic (ROC) curve analysis was used to determine the best interval of BMI and the quartile of FMI against two categories of PEI change: positive change (0) and the lack of positive change [1]. The cut-point (*c*), which is the optimal point for maximizing the number of correctly classified subjects, was calculated.

Area under the curve (AUC), which measures goodness of fit and validity of the model based on sensitivity and specificity, and the standard error (SE) were calculated. These values describe the ability of the test to detect the examined characteristic (sensitivity: the proportion of true positives that are correctly identified by the test) or to detect its absence (specificity: the proportion of true negatives that are correctly identified by the test).

The Youden index is defined for all points of an ROC curve, and the maximum value of the index may be used as a criterion for selecting the optimum cut-off point. The Youden index (*J*) was calculated as [24].

(2) $$\begin{array}{c} J=\text{maximum}\left\{\text{sensitivity}+\text{specificity}-1\right\},\text{ over all cut}‐\text{points}c,-\infty <c<\infty . \end{array}$$

Moreover, the *J* values were compared for successive BMI intervals and FMI quartiles to ascertain the optimal interval for the lowest risk of no positive change in PEI. A *p* value < 0.05 was considered statistically significant. All the calculations were carried out using Statistica 13.0 (StatSoft Poland 2018, Cracow, Poland).

## 3. Results

The boys were taller and heavier than the girls. The mean height for boys and girls was 176.46 ± 6.21 cm and 164.89 ± 6.08 cm, respectively, while body weight was 65.96 ± 14.38 kg for boys and 56.22 ± 7.82 kg for girls. Proportions of BMI and body composition, observed as FMI, were quite similar. The mean value of the BMI for the boys was 20.99 ± 4.00 kg/m^2^ and 20.62 ± 2.03 kg/m^2^ for girls, while the mean FMI was 8.87 ± 1.39 kg/m^2^ and 8.60 ± 1.76 kg/m^2^ for the boys and girls, respectively.

The relationship between sex and BMI, FMI, and PEI were statistically insignificant (*χ*^2^ = 5.83, *p* = 0.120; *χ*^2^ = 3.06, *p* = 0.383; and *χ*^2^ = 0.332, *p* = 0.564, respectively). Therefore, the boys and the girls were analysed together. The multiple regression analysis (not adjusted) showed that two variables, namely, BMI and FMI, explained approximately 19% of the variation in PEI before the intervention. Both indices (BMI and FMI) were found to be positively related to PEI, but the relationship was rather poor and statistically insignificant. FMI was the factor that influenced PEI the most (*β* = 0.188, *p* = 0.116; SE = 0.118; 95% CI 0.048–0.424), while the influence of BMI was very weak (*β* = 0.016, *p* = 0.892; SE = 0.118; 95% CI 0.219–0.251).

Table 1 shows the percentages of individuals without positive changes in PEI and with positive changes after the intervention using the BMI intervals and the FMI quartiles. There were no observed statistically significant differences (BMI: chi square = 2.459, *p* = 0.482; FMI: chi square = 2.272, *p* = 0.518). Across the first three BMI intervals, the percentage of individuals without positive effect in PEI was relatively low, ranging from 17% to 29%. In the last interval (>25 kg/m^2^), it increased to 43%. The calculated FMI quartiles were *Q*_1_ = 7.96, *Q*_2_ = 8.91, and *Q*_3_ = 9.65. The percentage of individuals with no positive effect in PEI for the FMI quartiles was quite different from that of the BMI intervals. Across the first three groups (below *Q*_1_, *Q*_1−2_, and *Q*_2−3_), the percentage increased from 16% to 37%, and in the last group (above *Q*_3_), it decreased to 28%.

Table 2 shows the *J* values for the BMI intervals and the FMI quartiles. *J* was the highest for BMI interval 19.01-22.00 kg/m^2^ and for FMI *Q*_2−3_. This means that the risk of lack of positive effects in PEI was the lowest for those ranges.

**Figure 1:**
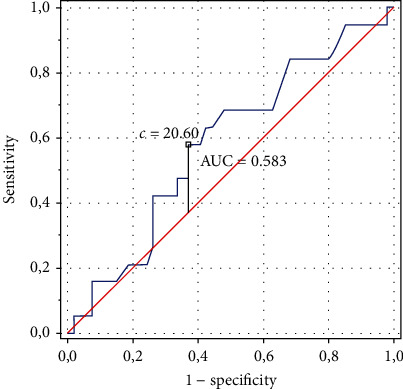
The receiver operating characteristic curve with optimal value of body mass index for the lowest risk of lack of positive changes in the physical efficiency index and area under the curve statistic to evaluate the quality of the performance of the model for body mass index.

**Figure 2:**
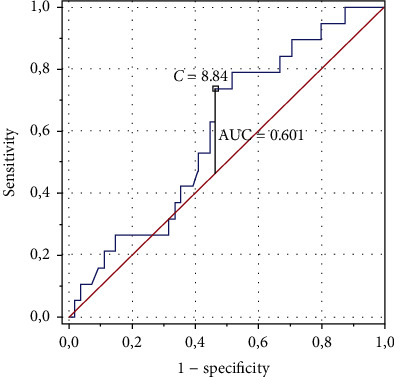
The receiver operating characteristic curve with optimal value of fat mass index for the lowest risk of lack of positive changes in the physical efficiency index and area under the curve statistic to evaluate the quality of the performance of the model for fat mass index.

Figures 1 and 2 show the ROC curves with optimal BMI and FMI values (respectively) for the lowest risk of lack of positive PEI effects. According to our data, the optimal BMI value was 20.60 and the optimal FMI value was 8.84 (Figure 1). The model for FMI was better adjusted (AUC = 0.601, *p* = 0.1544, SE = 0.071, 95% CI 0.462–0.741), while the model of BMI–worse (AUC = 0.583, *p* = 0.2700, SE = 0.075, 95% CI 0.436–0.7300) (Figure 2).

## 4. Discussion

The need for the analysis was highlighted by the lack of consistent physical efficiency results of HIIT programmes. General correlations among different HIIT programmes, motor performance, and physical efficiency were found by Huang and Malina [25]. Some authors observed positive results of HIIT in both normal weight and overweight children [12, 14], while others observed such effects only in children with a low BMI [15] and noted an improvement in cardiovascular parameters among overweight and obese children. These studies used HIIT programmes to induce changes in the body. Although BMI is frequently used to assess overweight and obesity, its usefulness is limited. This is because BMI does not always reflect true body fatness, and the ranges of the underweight, normal, and overweight intervals are wide [16].

Moreover, there is a problem with comparing the usefulness of the BMI and FMI in the assessment of health-related fitness of a population. Our research showed that including body mass components (fatty tissue) in such analyses enhances the value of the obtained results in comparison with analyses based on BMI alone. This applies to assessing the risk of obesity-related diseases in individuals with low muscle and high body fat, i.e., individuals with increased body fat and normal BMI [26]. Thus, the prediction of cardiovascular disease on the basis of BMI has limitations [17]. Therefore, extensive research has recently been devoted to the role of FMI [27, 28]. As part of the present research, the authors tried to establish the optimal cut-off points and their corresponding Youden indexes to distinguish individuals with regard to the lack of effects in physical efficiency after a 10-week HIIT programme. We aimed to determine the BMI and FMI ranges that predict the lowest risk of failure in the HIIT programme. Most studies have dealt with body size (BMI and FMI) as a determinant of health status, indicating, for example, the presence of metabolic syndrome [27, 29]. Studies on body size as a threshold for field-assessed aerobic performance are very rare [30], and ROC curves are usually used [31, 32].

In our study, the generated ROC curves showed acceptable AUC, suggesting that the calculated thresholds effectively distinguished between under- and overweight participants based on the HIIT physical efficiency effects. The threshold generated for FMI was slightly more accurate (AUC = 0.6) which means that FMI discriminates more efficiently (i.e., has a greater discriminating power) between adolescents; additionally, because it reflects true body fatness [16, 17], it is a key measure for the analysis. Our results indicated that the lowest risk of HIIT failure (the lack of effects after a multiweek intensive physical activity programme) was in the 19-22 kg/m^2^ BMI range (with the 20.60 cut-point) and in the 7.96-8.91 kg/m^2^ FMI range (with the 8.84 cut-point). This suggests that individuals even in the normal range of body mass, but close to its upper limit near overweight, do not experience a significant effect following a HIIT programme (a positive change after the 10-week programme). We do not yet have an explanation for this at this stage in our research.

We cannot directly compare our results with those obtained by other authors because of the lack of similar analyses. Boddy et al. [30] and Ruiz et al. [33] used body size parameters in relation to VO_2max_ consumption. In their studies, VO_2peak_ and VO_2max_ were estimated indirectly by means of a 20-minute multistage shuttle run performance (20-mSRT). Their results suggested better aerobic performance of youth with normal weight compared with overweight youth. This is consistent with our results. The participants classified as fit had significantly lower cardiometabolic risk scores than those classified as unfit [34].

This research is essential information for the activities of health care practitioners, teachers, and school administrators. BMI should not be the only measure of student obesity. FMI should also be assessed in cooperation with medical centres or institutions as part of public health.

Our results suggest that exercise programs need to be well designed by school administrators and consider educational systems; supervision of overweight, obese, and underweight students; enforcement and encouragement of physical activity; and enhancement of physical fitness levels. Creating student teams during PE classes that could implement Tabata exercise as an additional or alternative type of aerobic exercise has been suggested. For overweight and obese students, a good form for the activity would be additional school physical activities based on the Tabata program.

The limitations of this pilot study are as follows: small sample size and a limited number of sessions. Only one secondary school took part in the experiment, and there were only four intervals for BMI and FMI (owing to the small number of adolescents). In addition, there were only 10 sessions. Thus, purposive sampling with limited access to the study group decided about the lack of calculation of the statistic power analysis to determine the number sufficient to identify the effect size. Therefore, caution is required when interpreting and generalizing the results. It remains unclear whether the participants maintained their dietary preferences and what their energy supply was during the implementation of the 10-week HIIT (Tabata protocol) training program. In the future, participants should use accelerometers to monitor their daily physical activity.

## 5. Conclusions

These preliminary results enabled us to conclude that the optimal BMI value for the lowest risk of HIIT failure (no PEI effect) is 20.60 and the best BMI interval is 19.01-22.00 kg/m^2^. The value of 8.84 within range *Q*_2−3_ is optimal for FMI. A comparison of the two indices shows that FMI has stronger effects on PEI than BMI. In addition, the model obtained for FMI is characterized by higher accuracy. Further research is necessary to verify the above observations on a larger sample. The use of ROC generated cut-points by teachers and health promoters can be an alternative to the use of the BMI ranges in assessing health status. The FMI is particularly useful because it reflects true body fatness. Identifying at-risk individuals, or those in need of improving their health-related fitness (H-RF), and those with a low risk of poor H-RF allows for efficient planning of individual intervention services and training programmes. These issues are yet to be tested and should be considered in future investigations. We postulate continuing research with more adolescents from different socioeconomic environments and narrower intervals of the outcomes.

## Figures and Tables

**Table 1:** Distribution of youth with positive and lack of positive changes in the physical efficiency index (PEI) by BMI intervals and FMI quartiles.

BMI intervals (kg/m^2^)	Lack of PEI effect, *N* (%)	Positive PEI effect, *N* (%)	FMI quartiles (*Q*)	Lack of PEI effect, *N* (%)	Positive PEI effect, *N* (%)
≤19.00	3 (17.65)	14 (82.35)	Below *Q*_1_	3 (15.79)	16 (84.21)
19.01-22.00	11 (29.73)	26 (70.27)	*Q*_1−2_	4 (23.53)	13 (76.47)
22.01-25.00	2 (16.67)	10 (83.33)	*Q*_2−3_	7 (36.84)	12 (63.16)
>25.00	3 (42.86)	4 (57.14)	Over *Q*_3_	5 (27.78)	13 (72.22)

BMI: body mass index; FMI: fat mass index, *N*: number; %: percentage; PEI: physical efficiency index; *Q*_1−2_: between quartile *Q*_1_ and *Q*_2_; *Q*_2−3_: between quartile *Q*_2_ and *Q*_3_.

**Table 2:** The sensitivity and specificity for the risk of lack of positive changes in the physical efficiency index (PEI) by BMI intervals and FMI quartiles.

Body mass index (BMI)		Fat mass index (FMI)	
Intervals (kg/m^2^)	Youden index (*J*)	Quartiles (*Q*)	Youden index (*J*)
≤19.00	Reference groups	Below *Q*_1_	Reference groups
19.01-22.00	0.10	*Q*_1−2_	0.02
22.01-25.00	0.02	*Q*_2−3_	0.15
>25.00	0.08	Over *Q*_3_	0.03

*Q*_1−2_: between quartile *Q*_1_ and *Q*_2_; *Q*_2−3_: between quartile *Q*_2_ and *Q*_3_.

## Data Availability

Data are available upon request due to ethical restrictions regarding participant privacy. Requests for the data may be sent to the corresponding author.
